# Reply to Rattanapitoon et al., “Reconsidering the *Toxoplasma gondii* interactome: opportunities beyond crosslinking mass spectrometry”

**DOI:** 10.1128/mbio.02750-25

**Published:** 2025-10-09

**Authors:** Tadakimi Tomita, Elizabeth Weyer, Rebekah Guevara, Simone Sidoli, Jennifer T. Aguilan, Louis M. Weiss

**Affiliations:** 1 Department of Pathology, Albert Einstein College of Medicine 2006, New York, New York, USA; 2 Department of Biochemistry, Albert Einstein College of Medicine 2006, New York, New York, USA; 3 Department of Genetics, Albert Einstein College of Medicine 2006, New York, New York, USA; 4 Department of Medicine, Albert Einstein College of Medicine 2006, New York, New York, USA; Tsinghua University, Beijing, China

## REPLY

We thank Rattanapitoon and colleagues for their thoughtful comments on our study integrating crosslinking mass spectrometry (XL-MS) with machine learning to map a *Toxoplasma gondii* interactome. We appreciated their summary that “Tomita and colleagues (1) have presented a technically impressive map of the *Toxoplasma gondii* interactome, integrating crosslinking mass spectrometry (XL-MS) with machine learning. This study provides one of the most systematic attempts to probe protein–protein interactions in *T. gondii* and deserves recognition for bridging structural proteomics with computational modeling*.*” As detailed in our response in the following subheadings, we agree that the points that they raised will help sharpen the scope and future trajectory of this work.

## PROTEOMIC “VISIBILITY” AND STAGE-RESTRICTED BIOLOGY

We agree that our cytosolic preparation—performed without additional subcellular fractionation—prioritized abundant complexes (e.g., ribosome, proteasome) and that this limits sensitivity to low-abundance or stage-restricted proteins. In our paper (1), we explicitly acknowledged this design constraint and the value of adding fractionation or enrichment methods to broaden coverage. Our data reflect this bias: tachyzoite transcript abundance was among the most influential biological features in our model, consistent with the tachyzoite stage of sample preparation and the dynamic range challenge.

Although our main data set used unfractionated cytosol, we did evaluate a size-exclusion chromatography enrichment on an additional sample to improve the crosslinked:non-crosslinked ratio, illustrating that targeted fractionation can measurably enhance XL-MS signal. In future work, we concur with the letter’s suggestion to pair XL-MS with subcellular fractionation (e.g., hyperLOPIT-guided workflows) and targeted enrichments (e.g., BioID/AP-MS) to distribute abundant proteins and reveal lower-abundance interactions. We also endorse the proposal to extend XL-MS to bradyzoite biology; recent advances in myotube host systems now permit formation of large, mature cysts with thick walls and drug tolerance, creating realistic opportunities to apply XL-MS directly to cyst wall interactomes.

## MACHINE LEARNING: AVOIDING CIRCULARITY AND ENABLING DISCOVERY

Rattanapitoon et al. rightly caution that including features derived from resources such as STRING or hyperLOPIT can introduce circularity, potentially reinforcing well-studied assemblies. We noted this risk in our publication and proposed several countermeasures, e.g. stricter negative controls and orthogonal validation, to preserve sensitivity to novel interactions. In addition, we wish to point out the following clarifications that address comments in their letter.

Our LightGBM model described in our publication (1) used intrinsic XL-MS quality metrics for labeling (e.g., 1% FDR pairs as positives; >50% FDR as negatives) and emphasized crosslink-intrinsic features—spectral counts (CSMs), intralinks, and residue-pair multiplicity—shown by SHAP analysis to dominate predictions. While biological features (including STRING and hyperLOPIT agreement) contributed, they did not replace crosslink-intrinsic evidence.

Echoing the letter’s recommendation, we are prioritizing “leave-one-source-out” strategies (e.g., training without STRING/hyperLOPIT-derived signals), stratified feature down-weighting, and the incorporation of orthogonal, stage-specific information (e.g., perturbation transcriptomics and CRISPR fitness landscapes) as features and benchmarks in our refinements of our machine learning strategies.

## DENSE GRANULE (GRA) NETWORKS AND TRAFFICKING INTERMEDIATES

We agree with Rattanapitoon et al. that the prominent GRA subnetwork observed in “cytosolic” preparations is biologically intriguing. In our publication (1) we discussed that GRAs likely entered our preparation in small quantities within vesicles, consistent with the crosslinker’s membrane permeability, and the cytosol preparation method used in our workflow. At the same time, we do not exclude the possibility that XL-MS did capture transient trafficking intermediates. Indeed, our residue-level mapping resolved direct versus indirect contacts and suggested that GRA7 is a central hub, refining prior co-elution and co-IP-based networks (ToxoNET cluster #26). Going forward, our research plan includes (i) timed crosslinking coupled to invasion, (ii) fractionation/enrichment of dense-granule and parasitophorous-vacuole compartments, and (iii) application of the same XL-MS framework to mature bradyzoite cysts to test whether analogous GRA-like assemblies mark cyst wall formation and maintenance—precisely the clinically relevant space highlighted by Rattanapitoon et al.

## CONCLUDING REMARKS

We believe our publication (1) provides a reproducible XL-MS pipeline, a residue-resolved GRA network, and a transparent ML framework that collectively validate known assemblies and elevate true positives among mediocre-score hits. Coupling XL-MS to fractionation, bradyzoite systems, and orthogonal computational/experimental constraints will accelerate the transition from validation toward discovery in stage-specific, host-relevant biology. We agree with Rattanapitoon et al. that this will transform XL-MS from a methodological advance into a platform that shapes our understanding of host–parasite biology and drug development.
